# Individual Cortisol Production in Active Rheumatoid Arthritis Associates with Treatment Responses: A Pilot, Two-Year Study

**DOI:** 10.31138/mjr.131224.ipc

**Published:** 2025-05-28

**Authors:** Maria G. Filippa, Maria P. Yavropoulou, Nikolaos I. Vlachogiannis, George E. Fragoulis, Aimilia Mantzou, Aggeliki Papapanagiotou, Maria G. Tektonidou, George P. Chrousos, Petros P. Sfikakis

**Affiliations:** 1 First Department of Propaedeutic and Internal Medicine and Joint Academic Rheumatology Program, National and Kapodistrian University of Athens, Medical School, Athens, Greece;; 2 Endocrinology Unit, First Department of Propaedeutic and Internal Medicine, National and Kapodistrian University of Athens, Medical School, Athens, Greece;; 3 University Research Institute of Maternal and Child Health and Precision Medicine, National and Kapodistrian University of Athens, Medical School, Athens, Greece; 4 Department of Biological Chemistry, National and Kapodistrian University of Athens, Medical School, Athens, Greece

**Keywords:** saliva cortisol, rheumatoid arthritis, long-term remission, biomarker, response to treatment, glucocorticoids

## Abstract

**Introduction:**

Inadequate production of cortisol in relation to increased demands of chronic inflammation, a phenomenon coined as the “disproportion-principle”, occurs in some patients with active rheumatoid arthritis (RA). Moreover, relatively lower diurnal cortisol production prior to antirheumatic treatment initiation/escalation for active RA has been associated with inadequate corresponding treatment responses after 6-months.

**Objective:**

To evaluate whether individual levels of endogenous cortisol in active RA patients followed in an Academic Rheumatology Unit may predict the type of response to subsequent antirheumatic treatment regimens after two years.

**Methods:**

We measured morning circulating ACTH, cortisol and DHEAS blood levels, as well as saliva diurnal-cortisol levels (collected samples at 08:00, 12:00, 18:00, 22:00), prior to treatment initiation/escalation in RA patients with active disease. In a pilot study, we prospectively examined for possible associations between these measurements and treatment responses at two years in those 24 patients who were under optimal management according to standard protocols.

**Results:**

The ratio of circulating cortisol/ACTH, as well as diurnal cortisol production at baseline were significantly lower in patients with moderate response or no response to treatment (7/24, 29%), than in those having disease remission at two-years (17/24, 71 %). Baseline diurnal-cortisol-production greater than 81.3 (calculated as area-under-the-curve) could predict remission at 24 months with 86% specificity and 65% sensitivity, independently of age, sex and baseline CRP levels (p=0.03).

**Conclusions:**

Further studies to confirm that lower diurnal cortisol production prior to treatment initiation/escalation in patients with active RA may predict inadequate corresponding responses in the long-term, are warranted.

## INTRODUCTION

The efficacy of glucocorticoids (GC) in the management of rheumatoid arthritis (RA) and their beneficial effect on tissue damage was established through high-quality trials since late 50s.^1–4^ More recent longer-term follow-up studies have also shown that decreased progression of joint damage is maintained even after the glucocorticoid treatment has been discontinued.^5,6^ The most recent recommendations for the management of RA by the European Union Against Rheumatism (EULAR) acknowledge the fact that long-term administration of even low-dose glucocorticoids is frequently necessary to maintain clinical remission, even with the introduction of highly effective agents, such as the conventional disease-modifying anti-rheumatic drugs (cDMARDs), and biologics (b) or targeted-synthetic (ts) DMARDs.^7^

A compromised adrenocortical response to stress and inflammation, termed as “relative” or “functional” adrenal insufficiency has been previously described in some patients with RA, even in the absence of prior clinical and/or biochemical evidence of overt adrenal insufficiency,^8–13^ coined as the ‘disproportion principle’. As a result, endogenous adrenal cortisol production may be insufficient considering the increased clinical needs associated with the systemic inflammatory response, in at least a subset of patients with RA.^11,14,15^ Notably, both basal cortisol levels and time-integrated cortisol response to tetracosactide stimulation (Synacthen test) are significantly lower, compared to healthy controls, in patients with chronic inflammatory rheumatic diseases, who, while being in long-term remission with DMARDs combined with very low-dose GC, relapse upon discontinuation of GC, in the absence of adrenal insufficiency.^16^

In the current lack of relevant biomarkers with a predictive role for response to antirheumatic treatment in patients with RA we have previously shown that low basal morning saliva cortisol (<13.9 nmol/L) can predict inadequate clinical response after 6 months of treatment with 75% sensitivity and 92% specificity in a real-world clinical setting.^17^ However, the long-term potential predictive value of low cortisol levels concerning the clinical trajectory of rheumatoid arthritis has yet to be elucidated. Along these lines, herein we report the results of a pilot study to further investigate the relationship between baseline cortisol levels and clinical outcomes in rheumatoid arthritis over an extended period of 2 years.

## PATIENTS AND METHODS

The present study is a follow-up of our single-centre, prospective cohort study, conducted at the Rheumatology Unit of the first Department of Propaedeutic and Internal Medicine, of the Medical School, National and Kapodistrian University of Athens.^17^ The protocol was approved by the scientific committee of “Laiko” General Hospital (E. Σ 38/21-01-2021) and was registered in ClinicalTrials.gov (NCT05671627). Our 6 months results have been previously published. ^17^

Patients with RA fulfilling the 2010 American College of Rheumatology (ACR)/EULAR classification criteria^18^ with moderate/high disease activity (28-joint Disease Activity Score (DAS28)-ESR/CRP >3.2)^19,20^ had been initially enrolled. Patients who continued their follow-up in our academic centre up to 24 months from baseline are included in the present analysis. All patients were treated as per clinician’s judgement with any combination of cDMARDs, b/ts DMARDS, and glucocorticoids (≤15mg/day of prednisolone or equivalent) following the EULAR recommendations for RA management (2019 update).^21^

All of the core set variables were evaluated for each patient [i.e., swollen joint count (SJC), tender joint count (TJC), patient’s global assessment (PtGA), C reactive protein (CRP) and erythrocyte sedimentation rate (ESR)] and a DAS28–score was calculated. Response to treatment was based on the EULAR response criteria that classify patients as good, moderate, or non-responders, using the individual amount of change in DAS-28 and the level (low, moderate, or high) of DAS-28 reached.^22–24^

Remission was defined based on the Boolean 2.0 criteria (i.e., each of the 3 core set variables TJC, SJC, CRP (in mg/dl) must have a value of ≤1, and a PtGA cut-off of 2 cm).^25^ Complete medical history, current medication, and history of GC withdrawal were obtained from the Patient’s database in compliance with the General Data Protection Regulation policy of the hospital.

### Sampling procedures and measurements

At baseline, blood was obtained from RA patients at morning hours (8.00am) after an overnight fast, for the measurement of plasma adrenocorticotropic hormone (ACTH), serum C-reactive protein (CRP) and serum cortisol, as previously described.^17^Serum dehydroepiandrosterone sulphate (DHEAs) (μmol/L) was also retrospectively measured from stored serum samples, using an electrochemiluminescence ECLIA on cobas® e immunoassay analyser (reference values are given stratified by age).Saliva samples were obtained from patients with RA the next day at prescheduled time points, namely at 08:00, 12:00, 18:00 and 22:00, also as previously described.^17^

#### Statistical analysis

Shapiro–Wilk’s test was used to assess for normality of distributions and continuous variables are presented as mean ± standard deviation (SD). Differences in continuous variables between groups were assessed by independent samples t-test or the non-parametric Mann Whitney U test, when appropriate; categorical variables were assessed by two-tailed Fisher’s exact test. Serial measurements of saliva cortisol during the day were used to calculate the area under the curve (AUC) by integrating the values of the saliva cortisol over the prespecified time points (8.00-12noon–6pm-10pm). Time-integrated daily cortisol production was then calculated employing the integrated area under the curve using the trapezoidal method.^26^ Since AUC was calculated by integrating the values of the saliva cortisol over the prespecified time-range, saliva cortisol units were cancelled out and therefore AUC is expressed as a numerical value without any associated units.

We employed receiver operator characteristic (ROC) curve analysis to evaluate the predictive ability of baseline time integrated daily saliva cortisol production (AUC)to distinguish RA patients who attained treatment -induced remission at 24 months from those who did not. Logistic regression was used to examine the association between time-integrated cortisol production (below the ROC-defined cut-off value) at baseline and remission at 24 months while controlling for potential confounding effects of age, sex, and CRP levels at baseline. “Disturbed” cortisol rhythm, was defined as the lack of anticipated decline in saliva cortisol in at least one of the 3 following timepoints; i) from 8am to 12-noon, ii) from 12-noon to 6pm, and iii) from 6pm to 10pm.^16^

Statistical analysis was conducted with Stata v.13, SPSS v.27 and GraphPad Prism v. 7.05.

## RESULTS

There were 24 patients with RA (83.3% females, mean age: 55 ± 11.3 years), of whom 12/24 had been newly diagnosed at baseline and initiated treatment with DMARDs, while the rest were presented with worsening of disease activity and required escalation of treatment. Current medication consisted of cDMARDs (9/24, 37.5%), b/tsDMARDs monotherapy (3/24, 12.5%), or a combination of DMARDS (12/24, 50%), while 6/24 (25%) were also receiving GC (mean daily dose: 5mg of prednisolone). **Table 1** presents a comprehensive overview of the treatment modalities administered to enrolled patients throughout the 2-year follow up period, encompassing all therapeutic agents that were utilised on an individual basis during this time frame.

**Table 1.** Overview of treatment modalities administered to enrolled patients throughout the 2-year follow up period.

**Individual cases**	**DAS-28 Baseline**	**DAS-28 2 years**	**Response to treatment at 2 years**	**GC**	**Cumulative GC dose (mg)**	**cDMARDS**	**bDMARDS**	**tsDMARDS**	Steroid free*
Case 1	4.2	6.0	no	●	5115	●	●	● ● ●	No
Case 2	4.2	3. 8	no	●	540	●			Yes
Case 3	5.7	3.2	moderate	●	563	●			Yes
Case 4	5.7	4.4	moderate	●	1072	●			No
Case 5	5.5	3.8	moderate	-	0	●	● ● ●	●	NA
Case 6	5.0	3.8	moderate	●	1288	● ●	● ●		Yes
Case7	4.2	3.2	moderate	-	0	● ● ●			NA
Case 8	4.1	1.4	good	●	2250	● ●			No
Case 9	6.0	2.5	good	●	2294				No
Case 10	5.2	1.9	good	●	3196	●	● ●		No
Case 11	4.8	2.5	good	●	3674.5	● ●			No
Case 12	6.6	2.3	good	-	0	●		●	NA
Case 13	4.2	1.9	good	●	516	●			Yes
Case 14	6.4	2.0	good	●	573	●	●		Yes
Case 15	7.1	2.4	good	●	520	●		●	Yes
Case 16	4.0	2.5	good	●	417	●			Yes
Case 17	6.0	1.8	good	●	747	● ●			Yes
Case 18	6.6	1.8	good	●	731	●		●	Yes
Case 19	4.4	1.7	good	●	620	●			Yes
Case 20	5.2	1.8	good	●	250	● ●	● ●	●	Yes
Case 21	6.2	1.8	good	●	255	●			Yes
Case 22	7.2	1.5	good	●	148	●	●		Yes
Case 23	6.2	2.5	good		0	● ●			NA
Case 24	5.9	2.0	good	●	1187	●			Yes

Patients were classified as good, moderate, or non-responders using the individual amount of change in DAS-28 and the level (low, moderate, or high) of DAS-28 reached at 2 years of follow up according to the EULAR response criteria.^22–24^

● Reflects different agents of the same category; dose escalation is also included

* Steroid -free remission was defined as successful withdrawal of GC for >1 month prior to visit without disease flair reported or clinically documented by the treating physician.

GC: Glucocorticoids; DAS: disease activity score; DMARDS: Disease-modifying antirheumatic drugs; b: biologic; ts: targeted synthetic.

At 2 years of follow-up, remission, was achieved by 70.8% (17/24) of our study cohort, of whom 58.8% (10/17) were on remission already from 6 months of treatment and remained stable up to 2 years. Notably, of the 17 patients on remission, 13 (54.2%) were on steroid-free remission (defined as successful withdrawal of GC for >1 month prior to visit without disease flair reported or clinically documented by the treating physician). No differences in clinical or laboratory parameters recorded at baseline, including age, disease duration, seropositivity, CRP levels and DAS28 score were observed between those on remission (n=17) and those with moderate or no response to treatment (n=7) **(Table 2)**.

**Table 2.** Baseline characteristics and saliva cortisol levels in active Rheumatoid arthritis patients stratified according to treatment outcome at 2 years.

**Baseline clinical and laboratory parameters**	Patients on Remission* at 2 years (n=17)	Patients with moderate or no response to treatment ** at 2 years (n=7)	**P-value**
**Female sex, n**	13/17	7/7	0.283
**Age (years)**	56.7 ± 11.7	55 ± 11	0.747
**Disease duration (months)**	83± 120	52.9±52.1	0.494
**Seropositive RA, n**	8/17	3/7	1.000
**Serum CRP levels (mg/L) (min-max) (RV: 0–5 mg/L)**	37.7± 42 (0.5–153)	8.10 ± 7.7 (1.75–25)	0.087
**DAS28-CRP**	**5.2 ± 1.3**	**4.7 ± 0.7**	**0.479**
**Plasma ACTH levels (pmol/L) RV of the assay: morning measurements: 1.9–11.4 pmol/L**	3.9± 3.1	3.2± 1.4	0.964
**Serum Cortisol levels (nmol/L)**	**350±175**	**241±67**	**0.143**
**Serum cortisol / ACTH ratio**	105.1 ± 49.1	69.7 ± 20.4	0.047
**Serum DHEAS levels (μmol/L)**	3.7 ± 3.0	3.8 ± 3.0	0.977
**Saliva Cortisol, 8am (nmol/L)**	16.4 ± 8.3	14.2 ± 7.1	0.418
**Saliva Cortisol, 12-noon (nmol/L)**	6.3± 3	4.3 ± 1.7	0.166
**Saliva Cortisol, 6pm (nmol/L)**	5 ± 4.1	2.6 ± 1.9	0.065
**Saliva Cortisol, 10pm (nmol/L)**	4.5± 3.4	2.4 ± 4.5	0.114
**Time integrated daily saliva cortisol production - (AUC)**	99± 42	68 ± 19	**0.047**
**Disturbed cortisol rhythm n(%)**	10/17 (58.8%)	3/7 (42.9%)	0.659

* Remission was based on the Boolean 2.0 criteria.^25^

** Patients were classified as good, moderate, or non-responders using the individual amount of change in DAS-28 and the level (low, moderate, or high) of DAS-28 reached at 2 years of follow up according to the EULAR response criteria.^22–24^

RA: rheumatoid arthritis; ACTH: Adrenocorticotropic hormone; DHEAS: Dehydroepiandrosterone sulphate; AUC: area under the curve; CRP: C-reactive protein; GC: glucocorticoids.

We next investigated the potential predictive value of the baseline evaluation of i) diurnal production of saliva cortisol, ii) plasma ACTH and serum cortisol levels and iii) serum DHEAS levels in the long-term response of our RA patients. Time integrated daily saliva cortisol production at baseline was significantly higher in those patients on remission at 2 years compared to the rest of the patients (99± 42 vs. 68 ± 19, respectively, p=0.04). Individual saliva cortisol measurements at all time points during the day were higher in patients on remission at 2 years, albeit not reaching statistical significance. Circulating levels of ACTH, cortisol, and DHEAS did not differ between the two groups.

To further investigate the integrity of HPA axis in our patients we i) calculated the cortisol/ACTH ratio in a subset of patients (n=18), in whom both serum and plasma samples were available at baseline and ii) we evaluated the proportion of patients with disturbed saliva cortisol rhythm in the 2 groups. Patients on remission at 2 years demonstrated a significantly higher cortisol/ACTH ratio (14/18) compared to the patients with moderate or no response to treatment (4/18) (105.1 ± 49.1 vs. 69.7 ± 20.4 respectively, p=0.04), but the numbers were small. No significant difference was observed in the number of patients with disturbed cortisol rhythm at baseline between the 2 groups.

**Figure 1.**
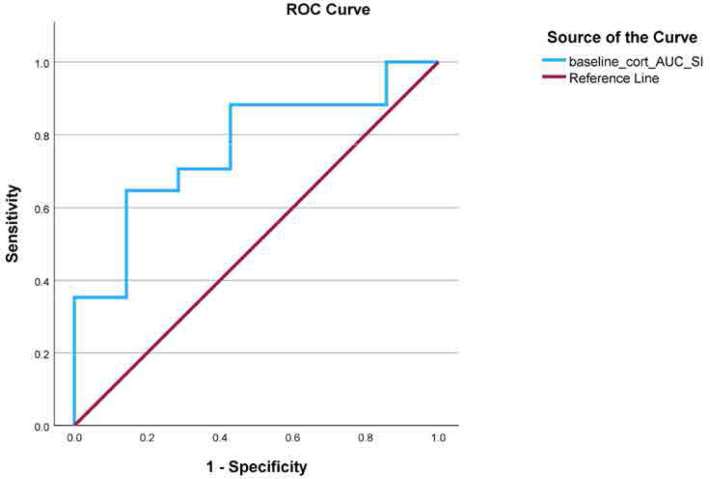
Receiver operating characteristic curve for baseline predicting disease-remission at 2 years of follow-up in patients with RA. Remission was **based on the Boolean** 2.0 criteria.^25^ AUC: area under the curve; DAS: Disease Activity Score; RA: rheumatoid arthritis.

Among all the parameters evaluated we found that time integrated daily saliva cortisol production (AUC) at baseline was significantly associated with attaining remission at 2years, independently of age, or baseline CRP levels (adjusted beta coefficient=3.83, SE=1.715, p=0.02). We next performed a ROC curve analysis to examine the ability of cortisol AUC at baseline to predict response to treatment after 2 years. A cut off AUC value ≥ 81.3 could identify RA patients attaining remission after 2 years of treatment according to EULAR recommendations, with 86% specificity and 65% sensitivity (Youden’s Index= 0.504, p=0.047) (**Figure 1).**

Steroid-free remission was reported in 13 out of 17 patients at 24 months **(Suppl. Table 1)**. Among the parameters evaluated at baseline, patients on steroid-free remission at 2 years had higher, albeit not-significantly, time integrated daily production of cortisol in the saliva and serum cortisol/ACTH ratio, but the numbers were small to draw any definite conclusions.

## DISCUSSION

In this study, we confirm the presence of a compromised adrenocortical functional reserve, as indicated by the lower baseline diurnal saliva cortisol production, in RA patients with moderate or no response to treatment at 2-years of follow-up despite being on highly effective DMARDs regimens. In the need of easily accessible biomarkers that could identify long term remission in RA, we suggest that a value ≥ 81.3 of time integrated daily saliva cortisol production (AUC) at baseline, can predict remission with 86% specificity and 65% sensitivity after 2 years of treatment initiation/escalation in RA patients. This is in accordance with our previous results where we showed that low morning saliva cortisol levels <13.9nmol/L, could be used to predict inability to achieve minimal disease activity after 6 months of treatment.^17^Moreover, we identified a significantly lower serum cortisol/ACTH ratio at baseline in RA patients with moderate or no response to treatment at 2 years compared to those on remission, mostly due to the lower serum cortisol levels, regardless of the provided treatment according to the international guidelines.^21^ Cortisol, as a stress hormone, is of pivotal value in inflammation restriction and its inadequate secretion when needed may have a negative impact in RA development, clinical outcome or long-term antirheumatic treatment response, as suggested in our pilot study. Clinical effectiveness of low-dose glucocorticoids in the management of RA has been reported from early years^1^ and their contribution in early and efficient control of Joint destruction has been well established.^4,27,28^ More recent multicentre randomised clinical trials have confirmed the beneficial effects of low-dose exogenous glucocorticoids over placebo or tapering in disease activity control in RA patients,^29,30^ despite the introduction into clinical practice of more targeted therapeutic agents. Therefore, glucocorticoids may be considered as a substitutional therapy for an impaired adrenal glands reserve in, at least some, patients with RA.^16,31^

On the other hand, although, significant progress has been made in our understanding of the cellular and molecular aspects of rheumatoid arthritis and regardless the utilisation of advanced machine learning, the lack of validated biomarkers that could predict treatment response remains an unmet need. In this context, several studies have attempted to identify circulating biomarkers in peripheral blood that could lead to a more precise treatment and a favourable clinical outcome, without adding, though, significant contribution in clinical practice.^32–34^ In the quest to identify circulating biomarkers of predictive value in RA, researchers are currently focusing on the molecular heterogeneity of the synovium tissue to target distinct molecular subtypes of the disease and to pave the way for precise medicine. As endogenous cortisol reduces inflammation by primarily acting through the glucocorticoid receptor (GR),^35^ the functional integrity of GR in the synovium tissue is currently under intense investigation.^36^ It has been shown that decreased cortisol production at the tissue level has been associated with higher GR expression in circulating leukocytes.^37^ In line with this evidence, using RNA-sequencing data from early and established RA, we demonstrated an increased expression of the GR in the synovial tissue of RA patients compared to healthy and osteoarthritic tissue.^38^ Higher GR expression could potentially serve as a compensatory mechanism that makes synovial tissue more sensitive to GC action in patients with disproportionally decreased cortisol in systematic circulation and explain the dramatically positive impact of exogenously-administered GC in RA management.^29–31^

Our pilot study has several limitations: i) the small sample size, since some patients were lost to follow-up, ii) patients with both early-stage and established RA were included in the analysis, increasing the heterogeneity of the study population and disease characteristics, iii) no patients with inactive disease at baseline were included as a control group, and iv) due to the non-interventional study design, as in our original cohort, no unified treatment protocol was used throughout the duration of the study, reflecting however, patients in the clinical setting of an Academic Rheumatology Unit. Taken together, our findings suggest that in a subset of RA patients there is a deficit in endogenous cortisol production that could affect disease pathophysiology and progression. Further studies are needed to confirm that low saliva cortisol levels at baseline in RA patients, could serve as potential valuable and easy-to-use bio-marker to predict treatment response and disease remission over long-term in routine clinical practice.

## AUTHOR CONTRIBUTION

Conceptualisation: M.P.Y and P.P.S. Data curation: all authors. Data analysis: M.G.F, N.I.V, M.P.Y, P.P.S. Funding acquisition: P.P.S. Investigation: All authors. Methodology: N.I.V, M.P.Y, M.G.F. Writing original draft: M.G.F and M.P.Y. Writing review and editing: all authors.

## FUNDING

No specific funding was received from any bodies in the public, commercial or not-for-profit sectors to carry out the work described in this article. Resources were used from ELKE grant 0974 (P.P.S.)

## CONFLICT OF INTEREST

All authors declare no conflict of interest relevant to this manuscript.

## NOTE

All authors take full responsibility for the integrity and accuracy of all aspects of the work. All authors confirm that the results of this follow up study have not been previously published as an abstract or in its full version anywhere else.

## ETHICS APPROVAL

The study was approved by the scientific committee of “Laiko” General Hospital (E. Σ 38/21-01-2021) and was registered in ClinicalTrials.gov (NCT05671627).

## PATIENT CONSENT

Obtained.

## Figures and Tables

**Supplementary Table 1.** Baseline characteristics and saliva cortisol levels in RA patients on remission (n=17), stratified according to successful steroids withdrawal at 2 years.

**Baseline clinical and laboratory parameters**	**Patients on steroid-free remission at 2 years (n=13)**	**Patients on remission with steroids at 2 years (n=4)**	**P-value**
Female sex, n	10/13	4/4	0.519
Age (years)	56.7 ± 13.1	56.75 ± 6.8	0.871
Diseaseduration (months)	62.2 ± 102	150.8 ± 163	0.296
Seropositive RA, n	6/13	2/4	1.000
Serum CRP levels (mg/L) (RV:0–5 mg/L)	45.7± 45	11.9 ± 9.1	0.163
DAS28-CRP	5.6 ± 1.2	4.1 ± 0.7	0.056
Plasma ACTH levels (pmol/L) RV of the assay: morning measurements: 1.9–11.4 pmol/L	3.7± 3.5	4.5 ± 2.4	0.374
Serum Cortisol levels (nmol/L)	352 ± 191	330 ± 141	1.000
Serum cortisol / ACTH ratio	116 ± 51.4	70.3 ± 14.9	0.217
Serum DHEAS levels	140 ± 123	142 ± 99	0.571
Saliva Cortisol, 8am (nmol/L)	18 ± 8.6	12.4 ± 6.5	0.296
Saliva Cortisol, 12-noon (nmol/L)	6.7 ± 3.3	4.9 ± 1.3	0.477
Saliva Cortisol, 6pm (nmol/L)	5.4 ± 4.4	3.9 ± 3.3	0.549
Saliva Cortisol, 10pm (nmol/L)	4.9 ± 3.7	3.1 ± 1.7	0.624
Time integrated daily saliva cortisol production - (AUC)	105.7 ± 43.8	75.2 ± 28.5	0.202
Disturbedcortisol rhythm n(%)	7/13 (53.8%)	3/4 (75%)	0.603

Steroid -free remission was defined as successful withdrawal of GC for >1 month prior to visit without disease flair reported or clinically documented by the treating physician.

RA: rheumatoid arthritis; ACTH: Adrenocorticotropic hormone; DHEAS: Dehydroepiandrosterone sulfate; AUC: area under the curve; CRP: C-reactive protein; GC: glucocorticoids.
